# Effect of Sedation on the Neurological Examination of the Patellar and Withdrawal Reflexes in Healthy Dogs

**DOI:** 10.3389/fvets.2021.664150

**Published:** 2021-05-10

**Authors:** Kristen T. Horsley, Natasha J. Olby, Mark A. Mitchell, Karanvir S. Aulakh, J. Alberto Gines

**Affiliations:** ^1^Department of Veterinary Clinical Sciences, School of Veterinary Medicine, Louisiana State University, Baton Rouge, LA, United States; ^2^College of Veterinary Medicine, North Carolina State University, Raleigh, NC, United States

**Keywords:** sedation, withdrawal reflex, dexmedetomidine, patellar reflex, neurological exam

## Abstract

**Introduction:** Pain, temperament, fear, and anxiety can prevent safe and accurate evaluation of common neurologic reflexes in dogs. When sedation is used it is unknown how the neurological examination, and specifically patellar and withdrawal reflexes are affected, and, if present, how long any effect might last. The purpose of this study is to investigate the effect of sedation on the evaluation of select common limb spinal reflexes in healthy dogs.

**Material and Methods:** Fourteen healthy dogs with normal neurologic exams were included. After placing joint landmarks, patellar reflex and pelvic and thoracic limb withdrawal reflexes were tested. Joint angles were measured, obtaining reflex angle endpoints, change in angle, and change in time to reflex completion. These measurements were recorded at different time points: prior to sedation (awake timepoint), 15 and 30 min following administration of standardized sedation protocol of dexmedetomidine and butorphanol, and 15 and 30 min following administration of a standardized reversal agent, atipamazole.

**Results:** For patellar reflex, the stifle end angle increased from 91.5 to 108.55 degrees (*p* < 0.0001) 15 min following sedation, and remained increased at 104.5 degrees (*p* < 0.0001) 30 min following sedation. Stifle change in angle increased from 9.6 to 24.4 degrees (*p* < 0.0001) 15 min following sedation, and remained increased at 20.85 degrees (*p* < 0.0001) and 11 degrees (*p* = 0.012) at 30 min sedation and 15 min reversal. Tarsal joint in pelvic withdrawal and elbow in thoracic withdrawal reflexes did not differ in at any timepoint of sedation or reversal when compared with the awake timepoint, for end angle or change in angle. The increases in end angle and change in angle for patellar reflex generated a change in time for patellar reflex from 0.12 s (awake) to 0.129 s (15 min sedation) which was statistically significant (*p* = 0.041). Change in time did not differ for pelvic withdrawal or thoracic withdrawal.

**Discussion/Conclusions:** Reflexes were elicited in all dogs under sedation. Sedation does not affect the evaluation of the withdrawal reflex on any limb but improves the visualization of the patellar reflex in this group of neurologically normal dogs.

## Introduction

Spinal reflexes are an integral part of a neurologic exam, and test the integrity of the sensory and motor components of the reflex arc and the influence of the descending motor pathways on that reflex (1, 2). The reflex arc represents a response after stimulus and the neurologic exam evaluates these spinal reflexes which help to identify damage to upper and lower motor neurons. Lesions can be localized to sections of the spinal cord (1). A reflex in the thoracic or pelvic limbs does not depend on the animal's conscious perception of the stimuli (3).

The patellar reflex is the most reliable myotatic reflex in the dog (4). The reflex is initiated by percussing the straight patellar ligament. Detection of the resulting muscle stretch leads to reflex contraction of the quadriceps femoris and provides information on the integrity of the L4-L6 spinal cord segments and the femoral nerve (3, 4).

The withdrawal reflex is a multi-segmental spinal cord reflex that plays an important role in the neurologic exam (3, 5). A noxious stimulus is applied to the tested limb by pinching the nail bed or digit with the fingers or a hemostat. The fourth digit of a limb, just proximal to the digital pad, has been identified as the most sensitive for manual application of noxious stimulus (6). Withdrawal reflexes provide neuro-localization information as part of the neurologic exams that are specific to the thoracic, C6-T2 spinal cord segment, and pelvic, L4-S2 spinal cord segment, limbs, respectively (3).

In a clinical setting, patients are often evaluated in lateral recumbency to facilitate visualization and consistency of the reflex. However, in some patients this can require significant restraint especially if the patient is anxious. When restrained, the patient can become more tense or excited, and this can prevent accurate evaluation of reflexes. Also, dogs often do not lie quietly especially when affected by extreme pain or are fractious (7). Because of pain, temperament, or fear, some patients cannot be safely examined or handled without restraint, and this can also avoid further injury to the animal (8, 9). Chemical restraint may be required to safely examine these patients, however general anesthesia will remove the veterinarian's ability to assess reflexes in a patient (10). Standardized sedation protocols have been established to aid in the physical exam assessment of patients, using drugs such as dexmedetomidine and butorphanol (8, 9, 11, 12). Sedation is commonly performed in practice, but it is unknown how the withdrawal and patellar reflexes are affected by sedation, and, if present, how long any effect might last.

The purpose of the study was to investigate the effect of a routine, established sedation protocol (8) on the neurologic examination of the spinal reflexes (patellar reflex, thoracic and pelvic limb withdrawal) in otherwise orthopedic and neurologically healthy dogs before, during, and after sedation by measuring the reflex angle endpoint, change in angle, and change in time to reflex completion. We hypothesized that the patellar and withdrawal reflexes would be decreased during sedation when compared to awake measurements, but would not be significantly decreased from the awake control following appropriate recovery from sedation measured at 30 min following reversal. The results of this study inform clinicians on how to interpret spinal reflex assessments on sedated dogs, additionally benefiting fearful or aggressive dogs who require a neurologic examination, and improve the ability to interpret neurologic examinations on dogs who have been sedated previously the same day.

## Methods and Materials

### Animals and Instrumentation

The study protocol was approved by the Institutional Animal Care and Use Committee (IACUC) at Louisiana State University (protocol number 19-066). Purpose-bred teaching and research Hound dogs were selected for participation in the study. Food was withheld the morning of each session. The dogs were considered to be healthy based on a physical exam, orthopedic exam, and neurologic exam. If any major orthopedic abnormalities that could affect a neurological examination or any neurologic abnormalities were identified, the dog was excluded from the study. All neurologic examinations were performed and reflexes tested by the same investigator (AG). The dogs were placed in lateral recumbency with the side being tested in the non-dependent position. The limbs were extended in a natural position without weight bearing or movement restriction of the non-dependent limb. The landmarks used for measurement on the pelvic and thoracic limbs were palpated and marked on the dog, according to the guidelines set by Fu et al. (13), with a colored sticker secured with glue (super glue; Gorilla Glue) to aid in consistent angle measurements of carpus, elbow, shoulder, tarsus, stifle, and hip. Upon completion of the experiments, the stickers and glue were removed with alcohol. The results were recorded on video and evaluated in slow motion. To ensure consistency in angle of video capture, the camera was held over the dog at a level of 120 cm high, centered on the stifle and elbow for the pelvic limb and the thoracic limb, respectively, and parallel to the dog in lateral recumbency.

### Sedation Protocol

The dog's vital parameters were measured which included cardiovascular status (heart rate), respiratory rate via auscultation, and non-invasive blood pressure using an appropriately sized cuff placed on a non-dependent forelimb with the dog in lateral recumbency. After baseline vital parameters and assessment measurements, described below, were recorded, each dog received the same sedation protocol. The sedation protocol consisted of intravenous dexmedetomidine 3 mcg/kg (Zoetis, Parsippany, NJ) and intravenous butorphanol 0.3 mg/kg (Zoetis, Parsippany, NJ) to mimic that used in a comparable clinical setting. The dogs were considered sedated when they remained in lateral recumbency and did not respond to an external stimulus handclap. The dog's sedation level was assessed and recorded using a previously validated twenty-point scale (Appendix 1) when dogs were awake, 15 min following administration of sedation, and 15 min following reversal (14–17). The dexmedetomidine was reversed with intramuscular (IM) atipamazole 0.03 mg/kg (Zoetis, Parsippany, NJ) following assessment under sedation. The dog was considered recovered from sedation when they were responsive to the surroundings and were able to walk unassisted. Vital parameter measurements were taken while the dog was awake, every 5 min while under sedation and, once reversed, at 15 and 30 min following reversal. If dogs had side effects or adverse reactions to sedation, they were excluded from the study.

### Assessment of Patella Reflex and Pelvic Limb and Thoracic Limb Withdrawal Reflexes

**Figure 1:**
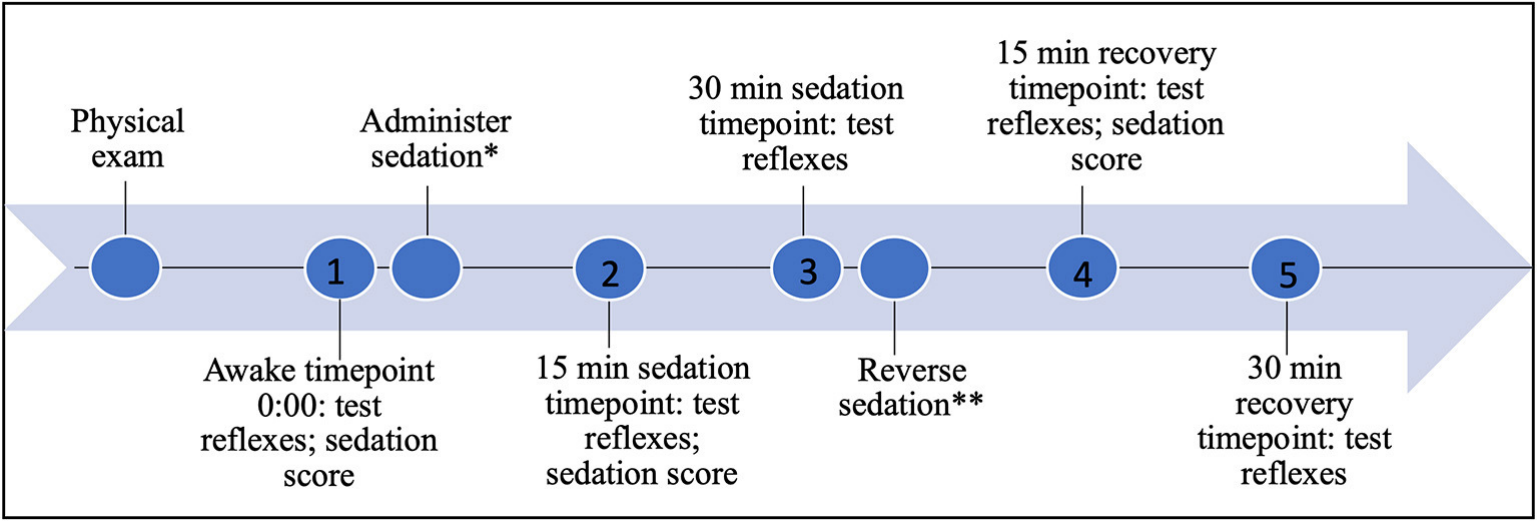
Timeline of testing, sedation, and recovery; *0.3 mg/kg butorphanol and 3 mcg/kg dexmedetomidine IV; **0.03 mg/kg atipamezole IM; **1** awake timepoint 0:00; **2** 15 min sedation timepoint 0:15; **3** 30 min sedation timepoint 0:30; **4** 15 min reversal timepoint 0:45; **5** 30 min reversal timepoint 1:00.

The patellar and withdrawal reflexes were recorded with a digital camera to evaluate in real time and slow motion the components of each tested reflex, the start and end angles of the joints during each tested reflex, and time from application of the stimulus to peak motor response to assess latency and magnitude (NCH software, videopad; Greenwood Village, CO). Using the start and end angles and time measurements, end angle, change in angle, and reflex duration were calculated. Horos software (Nimble Co LLC d/b/a Purview in Annapolis, MD USA) was used to measure the joint angles of the limbs and time to peak motor response. Both right and left limb patellar and pelvic and thoracic limb withdrawal reflexes were tested three times for each treatment timepoint. The following process was performed and recorded prior to sedation, referred to as “awake,” 15 and 30 min following administration of sedation, and 15 and 30 min following administration of the reversal agent (Figure 1). The side order (recumbency side of the dog) in which the reflexes were tested, was randomly assigned and remained consistent for each round of testing application, accomplished by means of an online statistical computer program (https://www.random.org/). The testing order of reflexes was consistent throughout the procedure: patellar, pelvic limb withdrawal, thoracic limb withdrawal.

The patellar reflex was tested with a reflex hammer by percussion of the patellar tendon between the patella and the insertion on the tibial tuberosity. To ensure consistency of stimulus intensity, the same investigator applied the reflex hammer stimulus (AG). The stifle was supported around 90 degrees flexion prior to the application of the stimulus, and the angle of the stifle at peak response was measured. Based on the definition of the patellar reflex resulting in extension of the stifle angle (4), the maximal stifle extension represented the end of the reflex for a peak motor response result.

**Figure 2:**
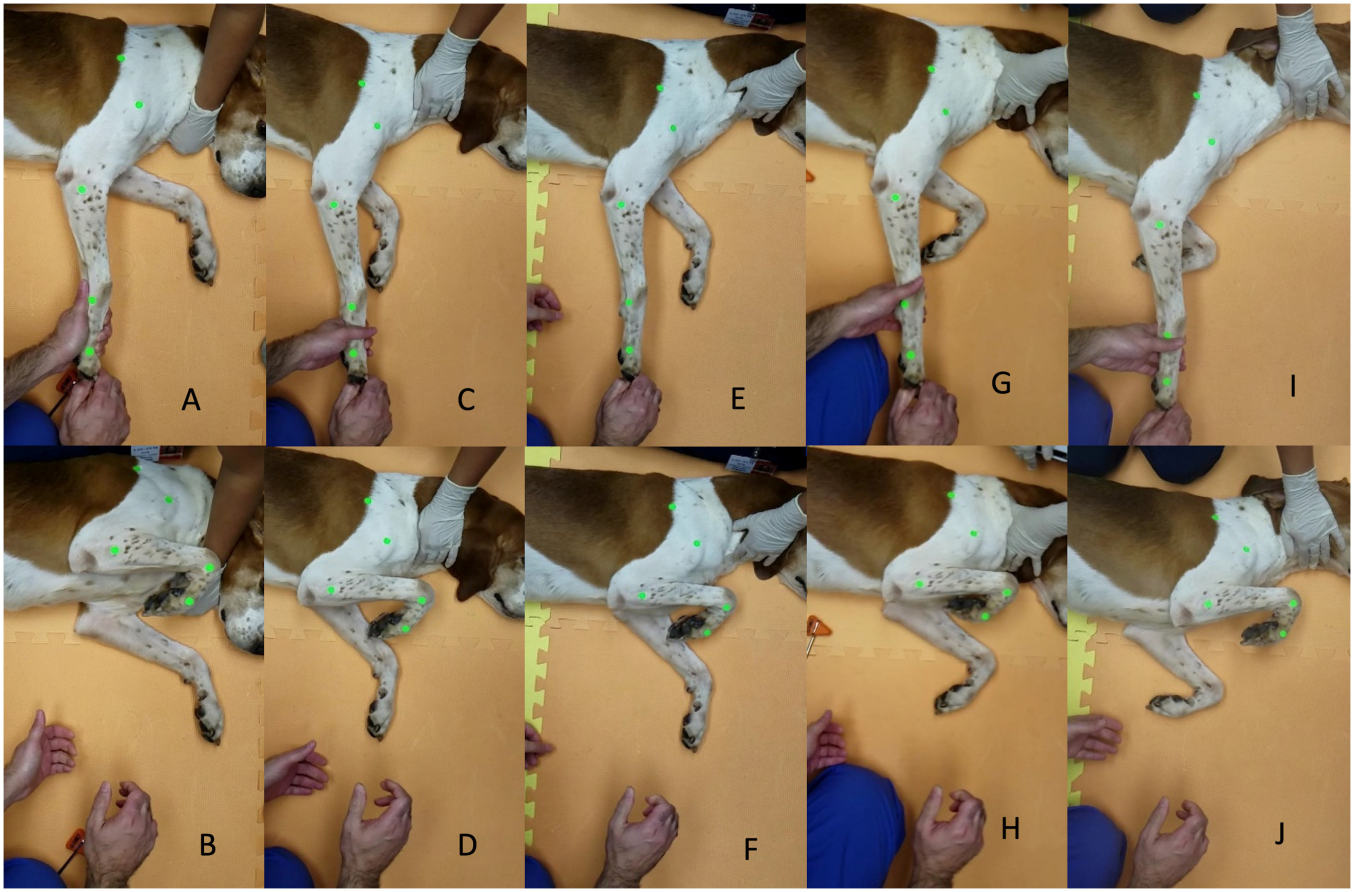
**(A–J)** Example of testing thoracic limb withdrawal at the start of the reflex and end of the reflex, respectively, for awake timepoint **(A,B)**, 15 min sedation timepoint **(C,D)**, 30 min sedation timepoint **(E,F)**, 15 min reversal timepoint **(G,H)**, and 30 min reversal timepoint **(I,J)**.

The withdrawal reflex was stimulated with digital pressure, a pinch, applied on the plantar or palmar aspect of the foot, respectively, proximal to the pad of the fourth digit with the limbs extended, as previously described by Hunt et al. (6). To ensure consistency of stimulus intensity, the same investigator applied a pinch stimulus (AG). Based on the definition of the withdrawal response resulting in flexion of the hock, stifle, and hip and resulting in the flexion of the carpus, elbow, and shoulder (3), the maximal flexion of all joints represented the end of the reflex for peak motor response result (Figure 2, thoracic limb withdrawal).

The patellar reflex, pelvic limb withdrawal, and thoracic limb withdrawal responses were each assessed in a combination of joints to reflect the overall reflex angle as it may be observable in a clinical setting in real time as well as at the level of the individual joint to determine whether a particular joint may be the sentinel marker for reflex completion and normalcy.

### Statistical Analysis

The Shapiro-Wilk test, skewness, kurtosis, and q-q plots were used to analyze the distributions of the data. Data that were not normally distributed were log-transformed for parametric analysis. For reporting purposes, normalized data are reported by the mean, standard deviation (SD), and range (minimum-maximum values), while non-normal data are reported by the median, 25–75 percentiles (%), and range. A mixed linear model was used to evaluate the effect of sedation on joint end angle. Dog was included in the model as the random variable and time as the fixed variable. Akaike's information criterion was used to assess the model. If a significance was found over time, a Bonferroni test was used to determine the specific differences between sample measurements. SPSS 24.0 (IBM Statistics, Armonk, NY, USA) was used to analyze the data. A *p* < 0.05 was used to determine statistical significance.

## Results

Of the 20 dogs available for the study, 14 met the inclusion criteria. Of the 6 dogs excluded, one had cardiac arrhythmia and five dogs had severe articular crepitus in different joints, probably due to osteoarthritis. Of the 14 dogs included in the study, there were 13 females and one male, with an average age of 2.8±1.3 years. The average weight of the dogs used in this study was 16.6±7.6 kg. No dogs had any adverse reaction to the sedation protocol used in this study. All the dogs included in the study were determined to have a clinically normal neurologic exam prior to inclusion.

Respiratory rate, heart rate, and non-invasive blood pressure were monitored prior to, during, and after sedation. The results are summarized in Table 1. The sedation score significantly differed between awake and sedated dogs at 15 min following sedation (*p* < 0.001), but did not significantly differ between awake and 15 min following reversal (Table 2). There was no significant difference in side order, therefore the side order did not alter the results for any angle or time measurement obtained.

**Table 1:** Vital parameters recorded at each timepoint during reflex testing.

**Vital parameter**	**Awake**	**15 min sedation**	**30 min sedation**	**15 min reversal**	**30 min reversal**
Respiratory rate^**^	23.64 (10.03)	10.14 (2)^*^	9.71 (2.59)^*^	10.86 (3.74)^*^	11.71 (3.5)^*^
Heart rate^***^	85.6 (14.8)	43.74 (9.42)^*^	48.31 (10.48)^*^	63.2 (11.94)^*^	62.2 (15.04)^*^
Systolic blood pressure^****^	132.81 (13.09)	108.61 (15.45)^*^	106.8 (16.4)^*^	113.16 (12.76)^*^	120.62 (21.18)

*Results are presented: mean (sd);*

* *significant (p < 0.05)*

** *breaths per minute;*

*** *beats per minute;*

**** *mmHg*.

**Table 2:** Sedation score^**^.

	**Awake timepoint**	**15 min sedation timepoint**	**15 min reversal timepoint**
Sedation score	0 (0)	16.57 (1.16)^*^	5.71 (2.92)

*Results are presented: mean (sd);*

* *significant (p < 0.05)*

** *see Appendix 1 for sedation score parameters*.

For the patellar reflex, the stifle end angle increased from 91.5° to 108.55° (*p* < 0.0001) 15 min following sedation, and stifle end angle remained increased to 104.5° (*p* < 0.0001) 30 min following sedation. However, the stifle end angle did not differ following reversal when compared to awake measurements (Table 3). The stifle change in angle increased from 9.6° to 24.4° (*p* < 0.0001) 15 min following sedation. Stifle change in angle remained increased to 20.85° (*p* < 0.0001) and 11° (*p* = 0.012) at each subsequent timepoint, 30 min following sedation, and 15 min following reversal, respectively (Table 4).

**Table 3:** End angle.

**Test**	**Joint**	**Awake**	**15 min sedation**	**30 min sedation**	**15 min reversal**	**30 min reversal**
Patellar reflex	Stifle	91.5 (81.53/99.68)	108.55^*^ (101.83/113.53)	104.5^*^ (99.65/110.38)	88.2 (76.7/99.68)	87.45^*^ (75.55/93.75)
Pelvic withdrawal	Tarsus	86.2 (76.53/96.75)	95.75 (85.5/104.83)	96.35 (86.3/108.35)	93.35 (85.45/103.43)	92.8 (82.08/101.25)
Thoracic withdrawal	Elbow	57.6 (45/67)	61.4 (50/72)	60.15 (50/72)	60.15 (52/66)	58.1 (51/67)

*Values reported as median (25/75%);*

* *significant (p < 0.05)*.

**Table 4:** Change in angle (absolute value).

**Test**	**Joint**	**Awake**	**15 min sedation**	**30 min sedation**	**15 min reversal**	**30 min reversal**
Patellar reflex	Stifle	9.6 (4.03/13.6)	24.4^*^ (18.1/29.5)	20.85^*^ (14.28/30.15)	11^*^ (4.75/15.15)	9.65 (5.3/14.4)
Pelvic withdrawal	Tarsus	62.75 (52.52/70.48)	53.55 (44.95/61.88)	51.75 (42.68/62.55)	53.45 (46.88/60.78)	53.25 (48.55/67.33)
Thoracic withdrawal	Elbow	83.85 (72.2/89.32)	77.55 (65.12/86.2)	76.6 (67/87.87)	81 (69.42/86.33)	81.65 (72.24/87.01)

*Values reported as median (25/75%);*

* *significant (p < 0.05)*.

For pelvic limb withdrawal, the tarsus did not differ in end angle at any timepoint of sedation or reversal when compared to awake measurements (Table 3). The tarsus change in angle was not different between awake and any timepoint following sedation or reversal (Table 4).

For thoracic limb withdrawal, the elbow end angle did not differ at any timepoint when comparing awake to sedation or reversal angles (Table 3). The elbow change in angle did not differ at any timepoint following sedation and reversal when compared to awake angle measurements (Table 4).

For patellar reflex, the change in duration of reflex measurements differed from 0.12 to 0.129 s (*p* = 0.041) between awake and 15 min following sedation. The remainder of the timepoints did not show any difference in change in duration for patellar reflex. There was not a difference between change in time for pelvic withdrawal and thoracic withdrawal reflexes (Table 5).

**Table 5:** Change in time.

**Test**	**Awake**	**15 min sedation**	**30 min sedation**	**15 min reversal**	**30 min reversal**
Patellar reflex	0.12 (0.09/0.14)	0.129^*^ (0.09/0.16)	0.122 (0.1/0.15)	0.111 (0.09/0.13)	0.108 (0.09/0.14)
Pelvic withdrawal	0.485 (0.38/0.64)	0.524 (0.41/0.66)	0.5 (0.4/0.6)	0.48 (0.38/0.61)	0.457 (0.38/0.52)
Thoracic withdrawal	0.505 (0.35/0.63)	0.533 (0.37/0.62)	0.477 (0.39/0.62)	0.506 (0.38/0.58)	0.47 (0.34/0.56)

*Values reported as median (25/75%);*

* *significant (p < 0.05)*.

## Discussion

In this study, reflexes were elicited in all dogs under sedation. The individual joint parameters measured for the patellar reflex, pelvic limb withdrawal, and thoracic limb withdrawal reflex were not significantly decreased at any timepoint during the study. The patellar reflex was shown to have significant increase in stifle angle, both end angle, and change in angle, under sedation. Therefore, the null hypothesis was partially rejected.

Vital sign measurements were obtained during the study period and correlated with sedation score. When sedated, the dog's sedation score increased indicating they were appropriately less likely to resist in lateral recumbency, and responded less to external stimulus clap noises both of which can contribute to stress and tense muscle posture; this was reflected through a significant decrease in measurable parameters of respiratory rate, heart rate, and systolic blood pressure. The sedation score was significantly different when comparing the awake timepoint to 15 min following sedation and 15 min following reversal. All dogs were reversed enough to walk back to their housing, but remained less reactive to loud external noise stimuli. This can be explained by residual effects from butorphanol. The half life is short, 1.7 h, and is not commonly reversed in a clinical setting. By not reversing butorphanol, the results reflect a persistent significant difference in change in angle of the stifle for patellar reflex when compared to awake measurements; similarly, respiratory rate and heart rate remained significantly different when compared to the awake timepoint.

Sedation significantly affects patellar reflex test end angle and change in angle in healthy dogs. The significant increase in endpoint angle and change in angle for patellar reflex at 15 and 30 min following sedation indicates that sedation can help improve the visibility of the reflex in this group of neurologically normal dogs. The alpha 2 adrenergic agonist inhibits the release of noradrenaline into the synaptic cleft modulating sympathetic nervous system resulting in myorelaxation (9). The dogs can then become calm enough to relax the muscle groups engaged in the reflex, whereas while awake in a restrained lateral recumbency, they are often too tense so that a reflex hammer cannot overcome the flexion of the stifle. This was subjectively noted during the study. The dogs often maintained a tense flexed position of the pelvic limb, and stimulation from the reflex hammer for patellar reflex in the awake dog stimulated a reaction of flexing the limb further away from the stimulus instead of the expected extension of the stifle joint by engaging the patellar tendon.

The tarsus did not show significant difference at any timepoint during the treatment groups for pelvic limb withdrawal end angle and change in angle. In clinical cases it is common to evaluate the pelvic withdrawal response by evaluating the tarsal flexion which appear to be appropriate in sedated and recently reversed neurologically normal patients. The elbow did not show significant difference at any timepoint during the treatment groups during thoracic limb withdrawal end angle and change in angle. In a clinical case, the elbow may be considered as the joint of focus during a neurologic exam in sedated and recently reversed neurologically normal patients.

The only significant difference in change in time was noted for the patellar reflex 15 min following sedation and by only 0.11 s. This is not a clinically recognizable difference at normal speed, and required slow motion detection to measure and determine significance. In a clinical setting, there is unlikely to be a notable difference in the time it takes for a reflex to occur under sedation, however this increase in time is related to the increase of the end angle and change in angle of the stifle. There is a higher excursion of the stifle when the patellar reflex is tested under sedation which increase the time for the reflex to be completed.

The noxious stimulus source can affect the withdrawal reflex obtained. One limitation of this study was that the stimulus was applied by the pinch of a hand from one of the researchers. Although the same researcher was used to apply the stimulus to a consistent location on the limb, the amount of force applied cannot be controlled or measured in the study's setting. Controlled force application may have created more consistent results, but the goal of this study was to mimic a clinical setting as closely as possible. Subjectively it was noted that during sedation, slightly more force was applied to the limb for thoracic withdrawal reflex on the first side tested for that treatment group. This is likely due to the decreased response to surroundings from sedation and that the dogs were not being disturbed except obtaining vital parameters for the first 15 min following sedation injection. All subsequent noxious stimuli at the same timepoint measurement achieved visual reflex at the time of first stimulus application. An additional limitation was the time that elapsed to collect all data for each timepoint. The reflexes were repeated three times with small wait times between repetitions. Following the first stimulus of each reflex, the dog did not have the same amount of time to return to the baseline deeper state of sedation prior to stimulus for the second and third measurements. Similarly, dogs were tested on the second side immediately after rotating recumbency, so no time allowed for the return to baseline sedation. It is not uncommon in a clinical setting that patients will be repositioned when sedated for procedures, and these patients may be more responsive to stimulus. Side order was not noted to create a significant difference of the angle and time variables of interest in this study.

In addition, dogs were subjectively noted to be slightly more responsive to the environment during reflex testing on the second side. Dogs were randomly assigned to begin reflex testing on the right or left side, and no difference was noted in tests performed on one side of the dog compared to the other. Order of reflex does not play a role in detecting significant difference in reflex outcome in this study. Another limitation is that this study was performed using a specific sedation protocol, and the results may be different with other protocols.

This study was completed in healthy dogs; these results may be different in a population of dogs with neurologic and/or orthopedic problems.

## Conclusions

In this population of healthy dogs, sedation does not decrease the ability to evaluate the patellar reflex, pelvic limb withdrawal, and thoracic limb withdrawal reflexes. Standard sedation protocol with dexmedetomidine and butorphanol commonly used in a clinical setting can improve visibility of patellar reflex in neurologically normal dogs.

## Data Availability Statement

The original contributions presented in the study are included in the article/Supplementary Material, further inquiries can be directed to the corresponding author/s.

## Ethics Statement

The animal study was reviewed and approved by Institutional Animal Care and Use Committee (IACUC) at Louisiana State University (protocol number 19-066).

## Author Contributions

KH and JG contributed to the study design, conducted the study and collected all data, participated in the preparation of the manuscript, and review of the manuscript prior submission. NO contributed to the study design, participated in the preparation of the manuscript, and review of the manuscript prior submission. MM conducted the statistical analysis and participated in the review of the manuscript prior submission. KA contributed to the study design and participated in the review of the manuscript prior submission. All authors contributed to the article and approved the submitted version.

## Conflict of Interest

The authors declare that the research was conducted in the absence of any commercial or financial relationships that could be construed as a potential conflict of interest.
