# Supplementary material for: Effect of Sedation on the Neurological Examination of the Patellar and Withdrawal Reflexes in Healthy Dogs
Source: Front Vet Sci. 2021 May 10;8:664150. doi: 10.3389/fvets.2021.664150 (PMC8143191; doi:10.3389/fvets.2021.664150)
Supplement: Supplementary file 1 [file Data_Sheet_1.docx]

**Appendix 1**: Composite descriptive sedation score *as seen in Grint et al adapted from Young et al (1990) and Kuusela et al (2000)

1. Spontaneous posture

- standing = 0
- tired but standing = 1
- lying but able to rise = 2
- lying but difficulty rising = 3
- unable to rise = 4

2. Palpebral reflex

- brisk = 0
- slow but with full corneal sweep = 1
- slow but with only partial corneal sweep = 2
- absent = 3

3. Eye position

- central = 0
- rotated forwards/downwards but not obscured by third eyelid = 1
- rotated forwards/downwards and obscured by third eyelid = 2

4. Jaw & tongue relaxation

- normal jaw tone/(strong gag reflex) = 0
- reduced tone, (but still moderate gag reflex) = 1
- much reduced tone, slight gag reflex = 2
- loss of jaw tone and no gag reflex = 3

5. Response to noise (handclap)

- normal startle reaction (head turn towards noise/cringe) = 0
- reduced startle reaction (reduced head turn/minimal cringe) = 1
- minimal startle reaction = 2
- absent reaction = 3

6. Resistance when laid into lateral recumbency

- much struggling, perhaps not allowing this positioning = 0
- some struggling, but allowing this positioning = 1
- minimal struggling/permissive = 2
- no struggling = 2

7. General appearance/attitude

- excitable = 0
- awake and normal = 1
- tranquil = 2
- stuporous = 3
